# Neglect of skin wounds and the risk of becoming a *Staphylococcus aureus* nasal carrier: a cohort study

**DOI:** 10.1186/s12889-015-2104-8

**Published:** 2015-08-05

**Authors:** Hagai Levine, Raid Kayouf, Vladislav Rozhavski, Tamar Sela, Inbal Rajuan-Galor, Anat Tzurel Ferber, Shiraz Yona, Olga Gorochovski, Tami Halperin, Michael Hartal

**Affiliations:** Braun School of Public Health and Community Medicine, Hebrew University-Hadassah, Jerusalem, Israel; Israel Defence Forces Medical Corps, Tel Hashomer, Israel; Department of Military Medicine, Hebrew University Faculty of Medicine, Jerusalem, Israel

**Keywords:** Prevention, Epidemiology, Wounds, Skin and Soft tissue infections, Staphylococcus aureus, Carriage, Young adults, Behavioral determinants

## Abstract

**Background:**

Nasal carriers of *Staphylococcus aureus* have an increased risk of acquiring skin and soft tissue infections, which could manifest as outbreaks, especially in crowded settings. Current prevention programs are ineffective, antibiotic resistance is rising and risk factors for becoming a carrier are incompletely understood. We aimed to examine whether a behavior, the neglect of skin wounds, is a risk factor for becoming a *Staphylococcus aureus* carrier during training.

**Methods:**

We conducted a field-based cohort study among male infantry trainees in three seasons in Israel during 2011–12. Participants underwent anterior nares cultures and answered structured questionnaires on potential risk factors on two occasions: before and 3 weeks after start of training (N = 542). Attitudes and practices toward neglect of skin wounds were defined as perseverance in training at all costs, despite having a wound. Samples were processed within 18 hours for identification of *Staphylococcus aureus.* Univariable and multivariable logistic regression analyses were performed to assess risk factors for becoming a carrier.

**Results:**

Carriage prevalence increased by 43.3 % during training, from 33.2 % to 47.6 % (*p* < 0.01). One-fourth (25.4 %) of those with a negative culture before training became carriers. None of the socio-demographic characteristics was a risk factor for becoming a carrier while the risk was lower in the winter (Odds ratio [OR] = 0.42; 95 % confidence interval [CI]: 0.23-0.78, *p* < 0.01) and spring (OR = 0.46; 0.26-0.81, *p* < 0.01) seasons compared to the summer season. Neglect of skin wounds in practice *and* attitude was a risk factor for becoming a carrier (OR = 2.40; 1.13-5.12, *p* = 0.02), as well as neglect in practice *or* attitude (OR = 1.86; 1.04-3.34, *p* = 0.04) compared to no neglect when controlled for season. The preventable fraction in the population attributed to neglect of skin wounds was 33 %.

**Conclusions:**

Neglect of skin wounds is an independent, common and strong risk factor for becoming a *Staphylococcus aureus* carrier during training. This preventable behavior should not be ignored and should be addressed in public health programs during training and in other settings. Further research on behavioral determinants of *Staphylococcus aureus* carriage and infection is warranted.

## Background

*Staphylococcus aureus* is both a human commensal and a pathogen frequently causing clinically important infections such as skin and soft tissue infections (SSTIs), as well as life-threatening infectious diseases including pneumonia and sepsis [1]. Sporadic and epidemic SSTIs, frequently caused by *S. aureus,* are common among young adults. The burden of morbidity is even higher in crowded settings with close contact and intensive physical activity, such as on sport teams and in military settings [2–4]. The optimal SSTIs prevention strategy remains unclear, antibiotic resistance is rising and current prevention programs are ineffective [5]. Although carriers can carry *S .aureus* in multiple body sites, the anterior nares of the nose are the most frequent site [6]. Nasal carriers of *S. aureus* have an increased risk of acquiring an infection [7, 8]. Longitudinal studies show that about 20 % (range 12-30 %) of individuals are persistent nasal carriers, 30 % (16-70 %) are intermittent carriers, and 50 % (16-69 %) non-carriers [9]. van Belkum et al. suggested that intermittent carriers and non-carriers belong to the same type of nasal carriers [10]. Risk factors for becoming a nasal carrier are incompletely understood, and the investigation of possible risk factors, such as the rarely studied behavioral risk factors, may be useful in developing new preventive strategies [9]. Higher carriage prevalence has been found among individuals engaging in activities leading to skin wounds and those with skin infections [9]. Carriage prevalence, both of methicillin-susceptible *S. aureus* (MSSA) and methicillin-resistant *S. aureus* (MRSA), may be even higher among soldiers [11].

SSTI outbreaks have been reported frequently in the Israel Defense Forces (IDF) over the last decade with attack rates of up to 95 % among groups of infantry trainees [12, 13]. This epidemic and sporadic illness has been associated with a high burden of lost training days, significant clinical complications and deaths of infantry trainees in recent years [14].

Against this background, we aimed to examine whether *S. aureus* carriage prevalence increases during infantry training and whether a behavior, the neglect of skin wounds, is a risk factor for becoming a *S. aureus* nasal carrier.

## Methods

### Study design, settings and population

We conducted this field-based cohort study in three consecutive cohorts, drafted to three different IDF infantry training bases in July 2011 (“summer”), November 2011 (“winter”) and March 2012 (“spring”). Participants, all young adult males, underwent anterior nares cultures and answered structured questionnaires on potential risk factors on two occasions: just before and three weeks after start of training. The study was population-based, with no exclusion criteria. However, candidates undergo medical screening, and individuals with underlying medical conditions that could affect the performance of an infantry soldier in field conditions are excluded from infantry training and hence from the study. During the first three weeks of training, soldiers lived under crowded communal conditions (12–16 persons per room). This period included basic military training, physical exercises, classroom lessons and a short but intensive training period conducted in the field.

The study was conducted by trained medical personnel from the Preventive Medicine Branch staff. Participants privately answered questionnaires in writing and were notified that data collected will not be known to base cadre and will not have any effect on their training or service. Military cadre were excluded from the study presentation and data collection in order to insure the participants' informed consent and to prevent any coercion or influence of commanders on their subordinates. Each participant signed an informed consent form before recruitment to the study. The study was approved and supervised by the IDF Medical Corps Institutional Review Board.

### Variables and data sources

Season (summer; winter; spring) was based on timing of data collection, and also represent training base. Participants gave information on history of skin infections, life-style and socio-demographic factors, such as smoking status (nonsmoker; current smoker), country of birth (native Israeli; immigrant), number of children in household (0; ≥1) and age. Data on antimicrobial therapy prescription between the two samplings was retrieved from the military computerized medical records.

Questions on attitudes before and during training (identical questions) and questions on practices during training were scored on a 1–5 scale and grouped for analysis into two categories: 1-3/4-5, as in a previous study [15]. Attitudes and practices toward neglect of skin wounds (no neglect; neglect) were defined as perseverance in training at all costs, despite having a wound. In infantry basic training settings, the implication is lack of any attention to the wound. Attitude question was: “If the skin is wounded during training (cuts/abrasions), it is very important to continue training *at all costs*, despite the wound". Practice question was: “I, personally, persevere in my training *at all costs*, even if my skin is wounded (cuts/abrasions)”.

In addition, care of skin wounds in attitude (appropriate care; inappropriate care) was based on the response to the question: "The immediate care of a skin wound includes washing, cleaning and disinfection".

Additional practices assessed included share of personal drinking cup and share of personal towel (no share; share).

### Laboratory procedures

The samplings were conducted in the same manner on two occasions: before and three weeks after start of training by a trained team of healthcare workers and followed a written protocol. Nasal samples, from both anterior nares, were collected using an AMIES applicator (Copan, Brescia, Italy) and cultured within 18 hours at the IDF Central Medical Laboratory according to standard protocols. Samples were plated on tryptic soy agar plates with 5 % sheep blood agar and on chromogenic plates CHROMagar StaphAureus/ CHROMagar MRSA (Hy-labs, Rehovot, Israel) at 35 °C. After a 48 hours incubation period, *S. aureus* was identified by colony morphology, DNAse and staphytect plus (oxoid) agglutination kit. Screening for MRSA was conducted using 30 μg Cefoxitin discs on Mueller- Hinton agar (Hy-labs) by disc-diffusion, according to the recommendations of the Clinical and Laboratory Standards Institute (CLSI) [16]. *S. aureus* isolates were preserved at −70 °C on Hy-transport medium (Hy-labs).

### Statistical methods

We calculated carriage prevalence by dividing the number of participants with positive *S. aureus* nasal culture by the appropriate denominator of valid samples. We calculated overall and season-specific prevalence, before and during training. We compared prevalence before and prevalence during training, overall and by season, using McNemar's test. We defined becoming a *S. aureus* carrier as having a negative culture before training and a positive culture during training and calculated the proportion who became carriers with 95 % confidence interval (CI). We performed univariable analyses to assess risk factors for becoming a carrier using *χ*^2^ tests. In order to assess dose–response pattern, we created two composite variables for neglect of skin wounds and assessed them in the multivariable logistic regression models:

1. Attitude before *and* attitude during training (no neglect before *and* during training; neglect before *or* during training; neglect before *and* during training).
2. Practice *and* attitude during training (no neglect in practice *and* attitude; neglect in practice *or* attitude; neglect in practice *and* attitude).

We calculated odds ratios (OR) and their 95 % confidence intervals (CI) as measures of association for relative difference. We calculated preventable fraction in the population as a measure of association for absolute difference, according to Levin’s Population Attributable Risk formula [(q_pop_-q_unexposed_)/q_pop_] [17]. We performed statistical analysis with SAS software, version 9.2 (SAS Institute Inc., Cary, NC, USA).

## Results

### Population characteristics

Of the 797 male recruits approached to participate in the study, 674 (84.6 %) agreed to participate and underwent sampling before start of training. Three weeks after start of training, 19.6 % were lost to follow-up and not included in the analysis, yielding the study population (N = 542). There were no differences in socio-demographic characteristics or carriage prevalence before training between study participants and those who were lost to follow-up, except for the lower proportion of current smokers among study participants (18 % vs. 26 %). Mean participant age was 18.9 years (SD = 1.1), and 95 % of participants were aged 18–20 (see Table 1).

**Table 1:** Characteristics of study population, before training (N = 542)

Characteristic^a^	N (%)
Males	542 (100)
Season	
Summer	187 (34.5)
Winter	170 (31.4)
Spring	185 (34.1)
Nonsmoker	343 (82.3)
Current smoker	95 (17.7)
Native Israeli	470 (87.9)
Immigrant	65 (12.1)
No children in household	190 (35.3)
≥1 child in household	349 (64.7)

^a^For all characteristics, data were available for >95 % of participants

### *S. aureus* carriage prevalence

*S. aureus* carriage prevalence before training was 33.2 % (180/542) and did not differ by season or socio-demographic characteristics (data not shown).

**Fig 1:**
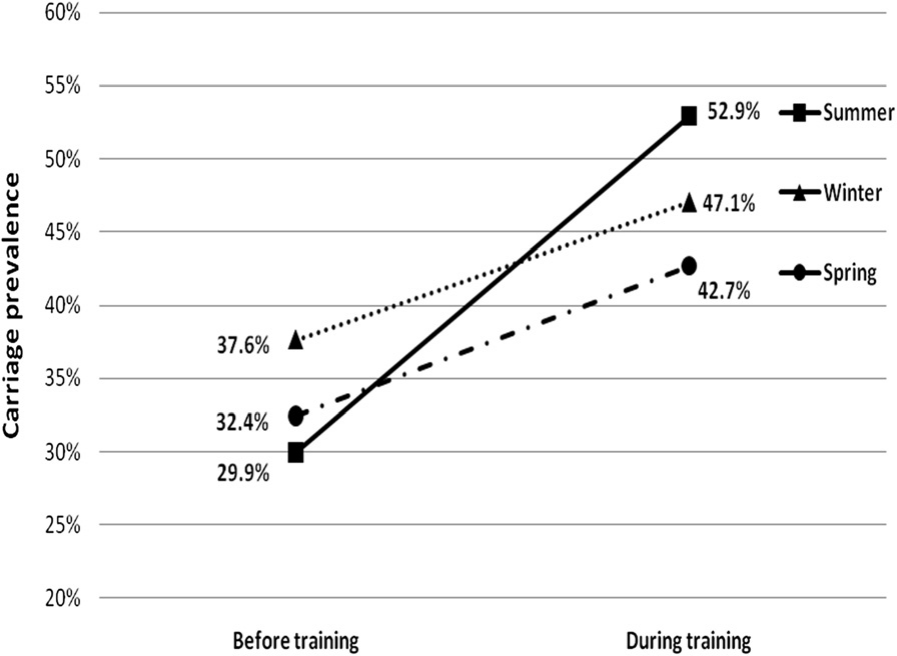
*Staphylococcal aureus* carriage prevalence by season, before and three weeks after start of training (N = 542)^a^. ^a^Increase in carriage prevalence during training was significant (p < 0.01) for each season

Carriage prevalence increased during training by 43.3 % (95 % CI: 24-66 %, *p* < 0.01), from 33.2 % to 47.6 %. In each of the three seasons, carriage prevalence increased during training (*p* < 0.01), most notably in the summer, by 76.8 %, from 29.9 % to 52.9 % (see Fig. 1).

Almost all (92.2 %, 166/180) carriers before training remained carriers, with similar proportions in the three seasons (summer: 53/56 = 94.6 %; winter: 59/64 = 92.2 %; spring: 54/60 = 90 %). A single isolate, cultured during summer, was identified as MRSA, while all other isolates were MSSA.

### Risk factors for becoming a carrier

Among 362 with a negative culture before training, 25.4 % (95 % CI: 21.1-30.1 %) became *S. aureus* carriers during training. Data on attitudes and practices were missing for seven participants, three of whom became carriers; yielding a sample of 355 participants for analysis of risk factors. Socio-demographic characteristics (smoking status, country of birth, number of children in household and age), history of skin infections and antimicrobial therapy prescription were not risk factors for becoming a carrier.

The risk of becoming a carrier was lower in the winter (OR = 0.42; 95 % CI: 0.23-0.78) and spring (OR = 0.46, 0.26-0.81) seasons compared to the summer season (see Table 2).

**Table 2:** Risk factors for becoming a *Staphylococcal aureus* carrier three weeks after start of training, univariable analysis (N = 355)

Risk factors	N (%)^a^	% carriers	OR (95 % CI)	p value
Total	355 (100)	25.1		
Season				
Summer	128 (36.1)	35.2	1.0	
Winter	102 (28.7)	18.6	0.42 (0.23-0.78)	<0.01
Spring	125 (35.2)	20.0	0.46 (0.26-0.81)	<0.01
Nonsmoker	287 (81.3)	25.4	1.0	0.84
Current smoker	66 (18.7)	24.2	0.94 (0.51-1.74)	
Native Israeli	310 (88.1)	23.9	1.0	0.18
Immigrant	42 (11.9)	33.3	1.59 (0.80-3.16)	
No children in household	127 (35.9)	26.8	1.0	0.54
≥1 child in household	227 (64.1)	23.8	0.85 (0.52-1.40)	
Attitudes before training				
Appropriate care of skin wounds	325 (92.3)	24.0	1.0	0.28
Inappropriate care of skin wounds	27 (7.7)	33.3	1.58 (0.68-3.67)	
No neglect of skin wounds	140 (39.6)	19.3	1.0	0.05
Neglect of skin wounds	213 (60.4)	28.6	1.68 (1.00-2.81)	
Attitudes during training				
Appropriate care of skin wounds	316 (89.3)	23.7	1.0	0.08
Inappropriate care of skin wounds	38 (10.7)	36.8	1.87 (0.92-3.81)	
No neglect of skin wounds	159 (44.9)	18.2	1.0	<0.01
Neglect of skin wounds	195 (55.1)	30.8	1.99 (1.20-3.30)	
Practices during training				
No neglect of skin wounds	77 (22.4)	16.9	1.0	0.05
Neglect of skin wounds	266 (77.6)	27.8	1.90 (0.99-3.63)	
No share drinking cup	261 (75.7)	27.2	1.0	0.78
Share drinking cup	84 (24.3)	21.4	0.73 (0.41-1.31)	
No share towel	64 (18.3)	20.3	1.0	0.35
Share towel	285 (81.7)	26.0	1.38 (0.71-2.66)	

^a^For all risk factors, data were available for >95 % of participants

Inappropriate care of skin wounds in attitude during training (11 % of the population) was a possible risk factor (OR = 1.87; 95 % CI: 0.92-3.81), and this subgroup had the highest risk of becoming a carrier (36.8 %).

Neglect of skin wounds in attitude before (60 % of the population) or neglect during (55 %) training was each a risk factor for becoming a carrier (OR = 1.68; 95 % CI: 1.00-2.81) and (OR = 1.99; 95 % CI: 1.20-3.30), respectively.

Neglect of skin wounds in practice (78 % of the population) was a possible risk factor (OR = 1.90; 95 % CI: 0.99-3.63). Those who did not neglect skin wounds in practice had the lowest risk of becoming a carrier (16.9 %), leading to a preventable fraction in the population attributed to neglect of skin wounds in practice of 33 %. In contrast, share of drinking cup or towel were not risk factors for becoming a *S. aureus* carrier.

We assessed correlations between neglect of skin wounds in attitudes and practices by Spearman correlation coefficients, which were 0.57 for practice and attitude during training (*p* < 0.01), and 0.42 for attitude before and during training (*p* < 0.01). Therefore, each time we re-introduced one risk factor (original or composite variable) into the multivariable logistic regression models, controlling for season which was the only significant covariate at a significance level of 0.05.

Risk factors magnitude and direction remained similar or was slightly accentuated after controlling for season (see Table 3). The composite variables exhibited a dose–response pattern, with gradient risk by having 2, 1 or 0 risk factors of neglect. Neglect of skin wounds in attitude before *and* during training was a risk factor for becoming a carrier (OR = 2.98; 95 % CI: 1.48-6.00), as well as possibly an attitude before *or* during training (OR = 1.68; 95 % CI: 0.95-2.96) compared to no neglect, controlled for season.

**Table 3:** Risk factors for becoming a *Staphylococcal aureus* carrier three weeks after start of training, multivariable logistic regression models, controlled for season (N = 355)^a^

Risk factors	OR (95 % CI)	p value
Attitudes toward skin wounds		
Appropriate care during training	1.0	
Inappropriate care during training	1.99 (0.97-4.08)	0.06
No neglect before training	1.0	
Neglect before training	1.84 (1.09-3.10)	0.02
No neglect during training	1.0	<0.01
Neglect during training	2.00 (1.20-3.34)	
No neglect before *and* during training	1.0	
Neglect before *or* during training	1.68 (0.95-2.96)	0.08
Neglect before *and* during training	2.98 (1.48-6.00)	<0.01
Practices toward skin wounds		
No neglect during training	1.0	0.05
Neglect during training	1.91 (0.99-3.70)	
No neglect, practice *and* attitude during training	1.0	
Neglect, practice *or* attitude during training	1.86 (1.04-3.34)	0.04
Neglect, practice *and* attitude during training	2.40 (1.13-5.12)	0.02

^a^Due to high correlation between attitudes and practices toward skin wounds, only one risk factor was introduced in each model

Neglect of skin wounds in practice *and* attitude during training, was a risk factor for becoming a carrier (OR = 2.40; 95 % CI: 1.13-5.12), as well as in practice *or* attitude (OR = 1.86; 95 % CI: 1.04-3.34) compared to no neglect, controlled for season.

## Discussion

In this study, *S. aureus* carriage prevalence among trainees increased drastically three weeks after start of training, reaching a prevalence of nearly 50 %. Almost all carriers before training remained carriers, while one fourth of those with a negative culture before training became carriers, with the highest risk in the summer. To the best of our knowledge, this cohort study is the first to discover that a behavior, the neglect of skin wounds, is a risk factor for becoming a *S. aureus* carrier. Neglect of skin wounds was an independent, common and strong risk factor, both in attitude and/or in practice, before and/or during training, with a dose–response pattern. The preventable fraction in the population attributed to neglect of skin wounds was 33 %, implying that prevention of this behavior could carry significant benefits.

The observed increase in *S. aureus* carriage prevalence during training was relatively high, increasing from 33 % to 47 %. MRSA was a rare finding in our population, as it was in another study among Chinese soldiers [18]. Nevertheless, the high prevalence of carriage and the fact that one fourth became a carrier of MSSA during training underscore the high risk in this crowded setting with close contact and intensive physical activity, and help explain previous reports of SSTI outbreaks during military training [19]. Military training is a risky period for bacterial transmission of other pathogens as well, as we previously shown for *Streptococcus pneumoniae* [15].

We discovered that neglect of skin wounds in practice and/or attitude is common among trainees and is a strong risk factor for becoming a *S. aureus* carrier. These findings are unlikely explained by chance, selection bias or differential information bias, given our study methods. Confounding by season or training base cannot explain the findings, as we controlled for these factors. As in any study, we cannot rule out residual confounding by other unknown factors. However, our findings appear to be specific to neglect of skin wounds and are not likely confounded by other behaviors, as no association was found with other behaviors such as smoking or sharing of drinking cup or towel. The relation between neglect of skin wounds and the risk of becoming a *S. aureus* carrier is further supported by temporality in our cohort study, strength of association, dose–response pattern and robustness of association across different measures for neglect of skin wounds, including attitudes and practices at various time points as well as another attitude toward appropriate care of skin wounds. These findings are plausible, taken previous observations of higher carriage prevalence among individuals conducting activities leading to skin wounds and those with skin infections [9]. The next step would be to replicate our findings in other populations and examine whether prevention programs addressing neglect of skin wounds are effective in reducing *S. aureus* carriage and burden of infectious diseases.

The prevention of SSTIs is complex and evidence for the effectiveness of commonly employed preventive measures is lacking. Current guidelines emphasize the importance of health education at the population level and heightened hygiene measures for individual patients with SSTIs, while less focus has been directed toward primary prevention efforts [20]. However, in a recent well-designed cluster randomized controlled trial, personal hygiene and education measures, including weekly use of chlorhexidine body wash, did not prevent SSTIs in general, nor MRSA SSTIs in particular, among a high-risk population of military trainees [5]. This study did not specifically address the issue of neglect of skin wounds. In contrast, retrospective surveillance analyses have shown a reduction of 30 % in SSTIs in a military training center following a comprehensive hygiene-based primary prevention program [21].

Our study suggests that summer season is a risk factor for becoming a *S. aureus* carrier during training. This finding reconfirms the established seasonality of SSTIs [22]. Each sampling season was conducted in a different training base and time, hence location or cohort effect, although unlikely, cannot be ruled out as a possible contributing factor. Season remains the most plausible explanation as we were unable to identify any substantial differences over time or in population characteristics (i.e. country of birth, number of children in household and age), behavior, training regimens or environmental conditions between the bases.

Our study has several limitations. First, the sample size was not powered to rule out weaker or less common risk factors (such as appropriate care of skin wounds). Second, only 80 % of the study population was available for follow up, raising the theoretical concern of a selection bias, unlikely in light of similar characteristics of those who were lost to follow-up. Third, our ability to identify carriage state was limited by having only two nasal cultures. Fourth, neglect of skin wounds was assessed by questionnaires and not direct observation, potentially biasing the results, i.e. due to social desirability. However, such potential bias would be non-differential and could only bias the results toward the null.

## Conclusions

Neglect of skin wounds is an independent, common and strong risk factor for becoming a *Staphylococcus aureus* carrier. This preventable behavior should not be ignored and should be addressed in public health programs during training and in other settings. Further research on behavioral determinants of *Staphylococcus aureus* carriage and infection is warranted.

## Abbreviations

CI: Confidence interval
IDF: Israel Defense Forces
MSSA: Methicillin-susceptible *S. aureus*
MRSA: Methicillin-resistant *S. aureus*
OR: Odds ratio
S. aureus: Staphylococcus aureus
SD: Standard deviation
SSTIs: Skin and soft tissue infections
Vs.: Versus
